# Renal Doppler as a Diagnostic Clue to Post-ductal Coarctation of the Aorta in an Adolescent Male: A Case Report

**DOI:** 10.7759/cureus.92289

**Published:** 2025-09-14

**Authors:** Mayank Srivastava, Namdev Seth

**Affiliations:** 1 Radiology, All India Institute of Medical Sciences, Gorakhpur, Gorakhpur, IND; 2 Radiodiagnosis, All India Institute of Medical Sciences, Gorakhpur, Gorakhpur, IND

**Keywords:** ct aortography, doppler ultrasound, endovascular management, tardus-parvus waveform, young hypertensive patient

## Abstract

Renal Doppler ultrasonography is a widely used, non-invasive modality in the evaluation of secondary hypertension, particularly in detecting renovascular causes such as renal artery stenosis. It was chosen as the initial modality in this case due to its non-invasive nature, absence of radiation, and established role in young patients with suspected secondary hypertension. However, it may also reveal indirect hemodynamic signs of proximal aortic obstruction. We report a rare case of a 15-year-old male who presented with unexplained systolic and diastolic hypertension and was subsequently diagnosed with post-ductal coarctation of the aorta, initially suspected based on bilateral tardus-parvus waveforms, an uncommon and often overlooked finding on renal Doppler. Further evaluation with CT aortography confirmed a post-ductal narrowing just distal to the left subclavian artery, with well-developed collateral circulation. Rather than being a novel function, the presence of bilateral parvus-tardus patterns illustrates the clinically valuable indirect role of renal Doppler in raising suspicion of upstream aortic obstruction. This case highlights the importance of considering coarctation in the routine workup of hypertensive adolescents and underscores the role of renal Doppler as an indirect but valuable diagnostic clue in extra-renal vascular abnormalities.

## Introduction

Coarctation of the aorta (CoA) accounts for approximately 5-8% of all congenital cardiovascular anomalies and shows a strong male predilection, with a male-to-female ratio of approximately 2-3:1 [1]. This condition is frequently associated with other congenital heart defects, such as bicuspid aortic valve, ventricular septal defect, patent ductus arteriosus, and complex syndromes like Turner, Shone, or PHACE. Clinically, CoA may remain silent until adolescence or adulthood, where it presents as systemic hypertension or its complications, including headaches, claudication, or stroke. Delayed recognition carries important prognostic implications, as untreated CoA is associated with persistent hypertension, left ventricular hypertrophy, aortic aneurysm, premature coronary artery disease, and increased cardiovascular morbidity and mortality. From an epidemiologic perspective, unrecognized CoA represents a preventable contributor to adolescent hypertension. Diagnosis is often suspected based on differential blood pressures between upper and lower extremities, or on incidental findings during evaluation for secondary hypertension [2].

Routine clinical examination or transthoracic echocardiography may fail to identify milder forms of CoA, particularly if femoral pulses are not carefully assessed or upper-lower limb pressures are not compared. Chest radiography and ECG reveal secondary changes only in advanced cases, while CT or MR angiography, though definitive, are not always used early due to cost, accessibility, or radiation concerns. In this context, renal Doppler ultrasonography is a preferred first-line tool for evaluating secondary hypertension in young patients, given its non-invasive nature, lack of radiation, and established utility in excluding renovascular disease. Beyond this role, it may also reveal indirect signs of proximal aortic obstruction. Bilateral tardus-parvus waveforms, although rare and easily overlooked, represent an important diagnostic clue, and their recognition is clinically relevant in hypertensive adolescents where coarctation may otherwise remain unsuspected [3]. We present a case where renal Doppler served as a critical diagnostic clue leading to the identification of post-ductal coarctation in an adolescent male.

## Case presentation

A 15-year-old male presented with intermittent occipital headaches for three months. He had no prior history of hypertension or familial cardiovascular disorders. On general examination, blood pressure measured 170 over 100 mmHg in both upper limbs, while lower limb blood pressure was significantly reduced at 110/70 mmHg, consistent with upper limb hypertension and lower limb hypotension, characteristic of post-ductal coarctation. On pulse examination, radial pulses were well felt, but femoral pulses were weak and delayed, with a clear radial-femoral delay. No bruits or murmurs were auscultated, and systemic examination was otherwise unremarkable. Routine laboratory workup, including complete blood count, renal and liver function tests, thyroid profile, and urine examination, was within normal limits. To investigate secondary hypertension, a renal Doppler ultrasound was advised.

**Figure 1:**
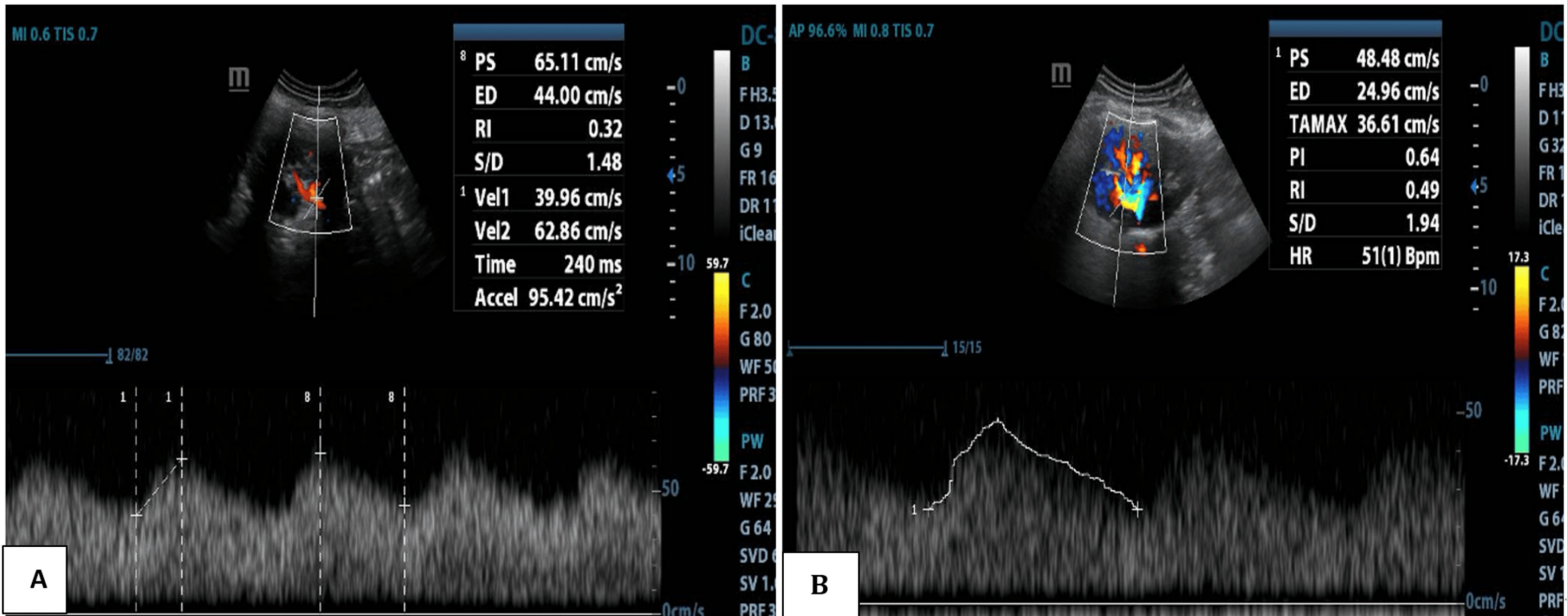
Renal artery Doppler of the main renal artery (A) and intrarenal arteries (B) demonstrating increased acceleration time, spectral broadening, and parvus–tardus waveform

**Figure 2:**
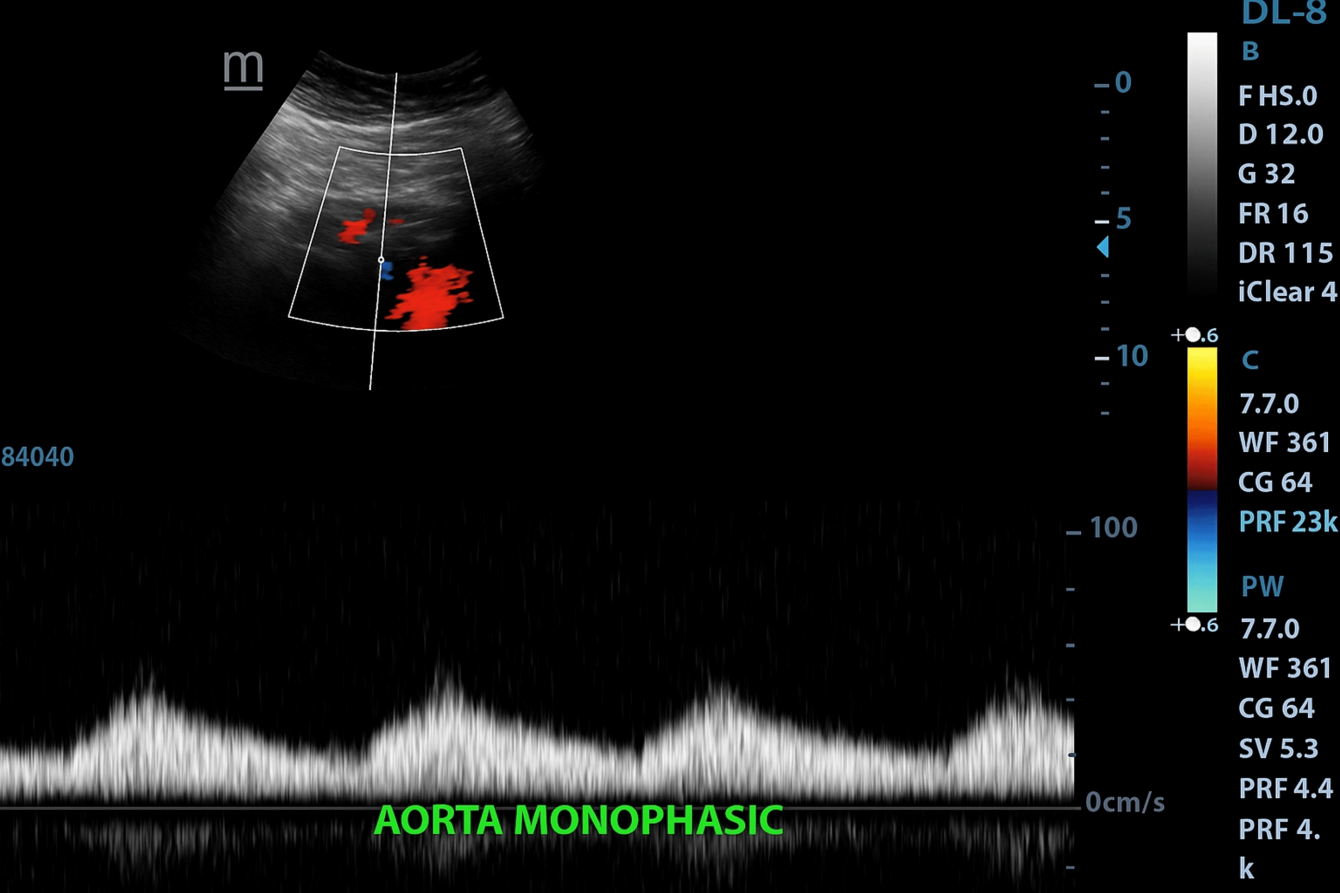
Color Doppler and spectral waveform analysis of the abdominal aorta demonstrating reduced peak systolic velocity and monophasic flow pattern, consistent with hemodynamically significant proximal obstruction

Renal Doppler revealed bilateral prolonged acceleration time ~240-250ms (Normal value~70ms) and spectral broadening in the main and segmental renal arteries, along with characteristic tardus-parvus waveforms (Figure 1). The abdominal aorta and bilateral lower limb arteries demonstrated reduced, monophasic flow (Figure 2), while the upper limb arteries maintained normal triphasic flow patterns. These findings were suggestive of significant proximal aortic obstruction.

**Figure 3:**
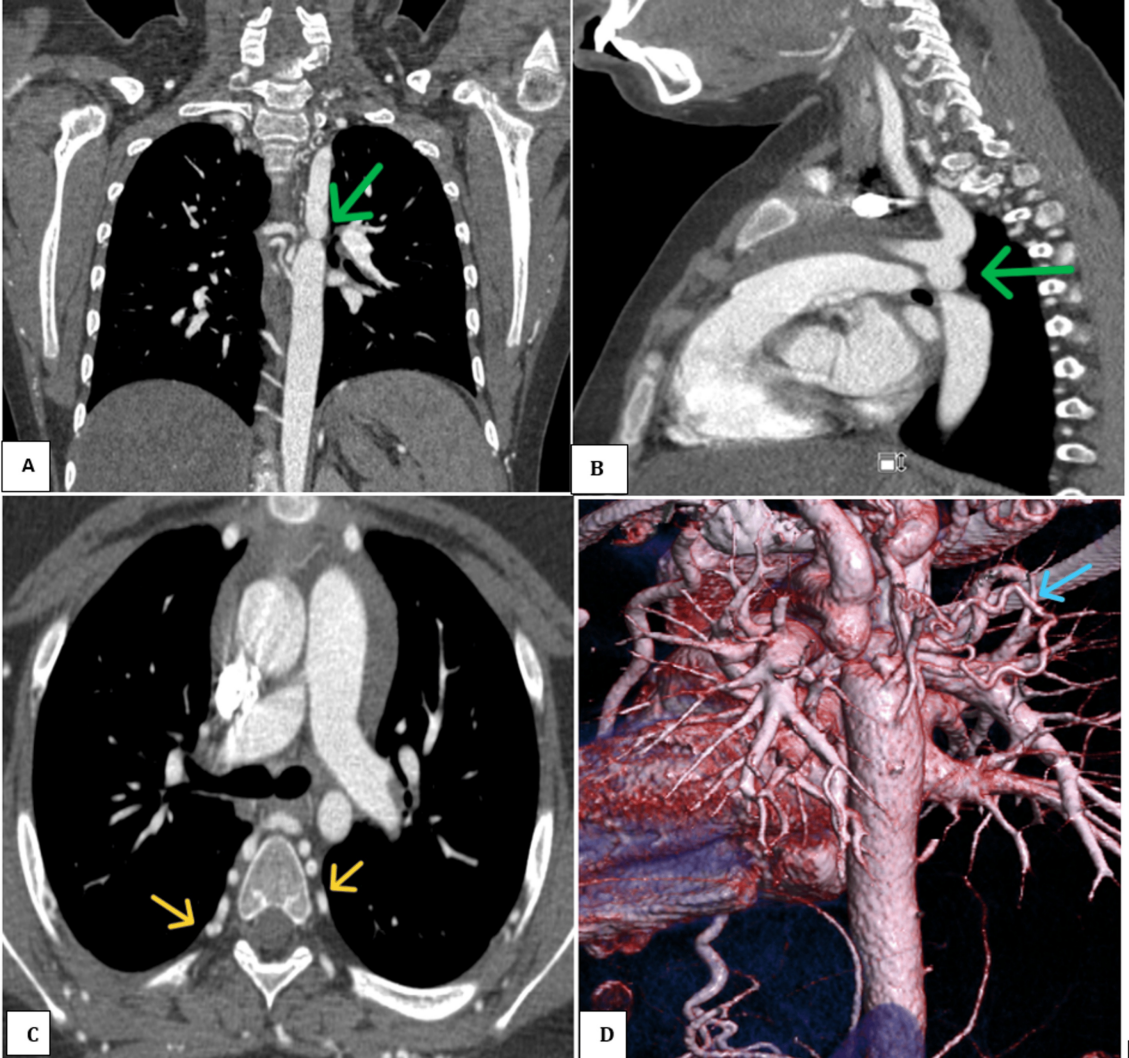
A and B: Coronal and sagittal sections of contrast-enhanced CT thoracic angiography showing a focal, post-ductal narrowing (green arrow) of the descending thoracic aorta just distal to the left subclavian artery. C: Axial section of contrast-enhanced thorax images showing multiple prevertebral collaterals (yellow arrow). D: Vascular reformat images showing multiple mediastinal collaterals (light blue arrow).

Transthoracic echocardiography was performed prior to CT to evaluate for associated anomalies, such as bicuspid aortic valve or septal defects; however, no intracardiac abnormalities were detected. Subsequent CT thoracic angiography was performed for further evaluation. It revealed a focal narrowing of the descending thoracic aorta just distal to the origin of the left subclavian artery, consistent with post-ductal (adult-type) coarctation. The narrowed segment measured approximately 8 mm in length with a minimal luminal diameter of 3 mm compared to 16 mm in the adjacent normal aorta. The ascending aorta showed pre-stenotic dilatation, and numerous arterial collaterals involving intercostal and paravertebral arteries were noted, without venous channels (Figure 3).

The final diagnosis was post-ductal coarctation of the aorta with collateral formation. Although interventional options, such as balloon angioplasty or stenting, were considered, the patient was referred for surgical repair due to limitations in endovascular facilities at our institute. Blood pressure was controlled medically in the interim, and the patient was referred to pediatric cardiothoracic surgery for further management.

## Discussion

CoA is a congenital narrowing of the aortic lumen that accounts for approximately 5-8% of congenital heart defects, with a male predominance [3]. Classification into pre-ductal (infantile) and post-ductal (adult) types is based on the relationship of the narrowing to the ductus arteriosus. The post-ductal form, as in the present case, more often manifests during adolescence or adulthood, commonly with systemic hypertension as the initial or sole clinical finding [4]. When physical signs, such as radio-femoral delay, murmurs, or differential limb blood pressures, are absent, the diagnosis may be delayed until complications such as left ventricular hypertrophy, premature coronary artery disease, cerebrovascular accidents, or aortic rupture occur. This absence of clinical findings may be explained by the development of extensive collateral circulation, which masks hemodynamic discrepancies despite significant underlying obstruction [5].

In our patient, a 15-year-old male with intermittent occipital headaches and hypertension confined to the upper limbs, clinical examination did not reveal murmurs, bruits, or other cardiovascular abnormalities. Routine biochemical evaluation was normal. The decision to investigate secondary causes of hypertension led to the performance of renal Doppler ultrasonography. Although Doppler sonography is most frequently applied to evaluate for renal artery stenosis, it can also reveal the indirect hemodynamic consequences of proximal aortic narrowing. In this case, both main and segmental renal arteries demonstrated markedly prolonged acceleration times of approximately 240-250 ms (normal <70 ms), spectral broadening, and the characteristic tardus-parvus waveform morphology. The abdominal aorta and bilateral lower limb arteries showed reduced monophasic flow, while the upper limb arteries maintained a normal triphasic pattern. This discordance strongly suggested a significant obstructive lesion proximal to the renal arteries.

The parvus-tardus pattern, characterized by a delayed systolic upstroke and diminished systolic velocity, reflects a severe upstream stenosis. When observed bilaterally in the renal arteries, particularly in the absence of local renal artery stenosis, it should prompt consideration of a thoracic aortic lesion, such as CoA, severe suprarenal abdominal aortic stenosis, large-vessel vasculitis (e.g., Takayasu arteritis), or surgical graft stenosis [6,7]. Previous case reports have described similar scenarios in which renal Doppler waveform abnormalities led to the first suspicion of CoA in adolescents and young adults with otherwise unremarkable physical examinations [8,9]. Bilateral renal artery tardus-parvus waveforms, combined with normal upper limb arterial flow, are considered highly suggestive of proximal aortic obstruction, and several authors have emphasized the utility of Doppler in this indirect diagnostic role [10].

Subsequent CT aortography in our patient confirmed a discrete narrowing of the descending thoracic aorta just distal to the left subclavian artery, with pre-stenotic dilatation of the ascending aorta and extensive mediastinal and paravertebral collaterals, confirming the chronicity of the lesion. The narrowed segment measured approximately 8 mm in length with a minimal luminal diameter of 3 mm compared with 16 mm in the adjacent normal aorta; collaterals were arterial in nature without venous channels. The absence of associated cardiac or renal anomalies was noteworthy. Although echocardiography is generally prioritized in pediatric populations to evaluate for associated anomalies, it was not the initial investigation in this adolescent, as the presentation was isolated hypertension, for which renal Doppler was performed first. Echocardiography was subsequently done and was unremarkable. These findings illustrate the complementary roles of non-invasive Doppler assessment and definitive cross-sectional angiographic imaging in diagnosing CoA. In adolescents, MRI is often preferred due to the lack of radiation and excellent functional assessment, while CT is faster, widely available, and provides superior spatial resolution of collaterals and calcification. In our case, CT was chosen based on availability and rapid turnaround [11].

Our case emphasizes the importance of considering CoA in the differential diagnosis of young patients with hypertension, even in the absence of classic clinical findings. Careful analysis of systemic and renal artery Doppler waveforms can provide crucial indirect evidence of proximal aortic narrowing, prompting timely confirmatory imaging. Long-term follow-up after repair is critical, as patients remain at risk of re-coarctation, aneurysm formation, and persistent hypertension despite successful correction. Earlier and routine blood pressure screening in adolescents may also facilitate earlier diagnosis, preventing delay until complications develop. Early recognition is vital, as surgical or endovascular correction significantly improves long-term survival and reduces the risk of late cardiovascular morbidity [5,11].

## Conclusions

This case underscores the utility of renal Doppler in detecting extra-renal vascular anomalies, such as coarctation of the aorta, especially in pediatric and adolescent patients with unexplained hypertension. The rarity of CoA being first suspected on renal Doppler highlights the novelty of this observation. In this patient, renal Doppler provided an indirect hemodynamic clue rather than a definitive diagnosis, which required confirmatory CT angiography. It should therefore be considered a useful adjunctive tool in the diagnostic algorithm of secondary hypertension. From a broader clinical perspective, the case emphasizes the importance of incorporating coarctation into the routine workup of adolescent hypertension. Echocardiography remains complementary in accordance with guidelines, particularly for assessing associated cardiac anomalies, and should be part of the complete evaluation. We acknowledge that our report is limited by the absence of invasive pressure gradients and complete Doppler parameters; nevertheless, it illustrates how indirect clues on Doppler can guide further targeted investigations. Finally, long-term follow-up after surgical or endovascular intervention is essential, given the risks of re-coarctation and persistent hypertension, reinforcing the clinical importance of timely recognition and appropriate management.
